# Systematic analysis and case series of the diagnosis and management of trichilemmal carcinoma

**DOI:** 10.3389/fonc.2022.1078272

**Published:** 2023-01-13

**Authors:** Jiachen Sun, Lihua Zhang, Minglu Xiao, Shiyi Li, Runkai Chen, Ying Li, Yuguang Yang

**Affiliations:** ^1^ Department of Dermatology, Fourth Medical Center of Chinese PLA General Hospital, Beijing, China; ^2^ Department of Pathology, Fourth Medical Center of Chinese PLA General Hospital, Beijing, China; ^3^ Department of Burns and Plastic Surgery, Fourth Medical Center of Chinese PLA General Hospital, Beijing, China; ^4^ Department of General Surgery, First Medical Center of Chinese PLA General Hospital, Beijing, China

**Keywords:** trichilemmal carcinoma, epidemiology, Mohs micrographic surgery, metastasis, recurrence, immunohistochemistry

## Abstract

**Background:**

Trichilemmal carcinoma (TLC) is a rare malignant cutaneous adnexal neoplasm, with no relatively comprehensive research.

**Objective:**

The aim of this study is to perform an updated statistical analysis so as to better understand TLC’s epidemiology, clinical features, diagnosis, and treatment.

**Methods:**

The diagnosis and treatment of three TLC cases in our department were summarized. Then, all TLC cases published in the literature were retrieved for a comprehensive analysis, followed by the analysis of global trends and regional distribution, demographic characteristics, clinical features, pathogenesis, histopathological features, and treatment and prognosis of TLC.

**Results:**

Of the 231 cases, the incidence of TLC has shown an upward trend recently, especially in China, in Asia. The susceptible population is men aged 60–80 and women over 80, and the most prone location is head and neck. The phenotype of TLC is not always typical and may be misdiagnosed because of the coexistence of other diseases. There is a linear relationship between the diameter and its duration or thickness. UV, locally present skin lesions, trauma, scarring, organ transplantation, and genetic disorders may trigger the occurrence of TLC. Periodic acid–Schiff staining and CD34, but not Epithelial Membrane Antigen (EMA), were helpful in the diagnosis of TLC. Although effective, surgical excision and Mohs micrographic surgery need further improvement to reduce recurrence of TLC. Carcinoma history is an independent risk factor for TLC recurrence.

**Limitations:**

The limitation of this study is the lack of randomized controlled trial on TLC treatment and recurrence.

**Conclusion:**

TLC has the possibility of invasive growth and recurrence, especially in patients with longer duration and carcinoma history.

## Introduction

As first described and defined by Headington, trichilemmal carcinoma (TLC) is a rare malignant adnexal neoplasm and exhibits features of “outer root sheath differentiation and atypical clear cell neoplasm” (1, 2). Clinically, TLC usually manifests as an asymptomatic exophytic or polypoid masses and may be misdiagnosed as basal cell carcinoma (BCC), squamous cell carcinoma (SCC), keratoacanthoma, or proliferating pilar cyst (3). Generally manifested as an indolent course, TLC may still be locally destructive and may have the possibility of recurrence or metastasis, which implies the significance of accurate diagnosis and careful management.

Given the fact that there was still no relatively comprehensive research on TLC, so on the basis of the questions that we encountered during the diagnosis and treatment of three TLC cases, we further retrieved all TLC cases in the published literature for a comprehensive analysis, aiming to provide a more thorough understanding of TLC. Starting with the global prevalence of TLC over time and the regional distribution, we summarized the demographic characteristics of TLC, including the analysis of age distribution and susceptibility location. The clinical features of TLC were further systematically exhibited, from the possible concomitant diseases to the size and duration of TLC with the potential associated factors analyzed. To explore the possible pathogenesis, the etiology of TLC was systematically summed up, which may have a prompting effect on the prevention of TLC. In view of the diverse manifestations of TLC, which may lead to the misdiagnosis or missed diagnosis, we gathered the pathological features of TLC and proposed key pathological indicators that may be helpful for the differential diagnosis of TLC *via* the statistical analysis of the staining results of all cases. Various treatment methods for TLC were summarized, and the recurrence-related risk factors were analyzed, which may provide a basis for improving the prognosis of patients with TLC.

Here, we reported three cases of TLC treated in our department. Then, we summarized all reported cases in literature so as to better understand the epidemiology, clinical features, diagnosis, and treatment of TLC. The comprehensive analysis of TLC has good auxiliary significance for the prevention, diagnosis, treatment, and prognosis of TLC.

## Materials and methods

### Ethics and specimen acquisition

The study was approved by the Ethical Review Committee of Fourth Medical Center of PLA General Hospital. Written informed consent was signed after the patients were being informed of the research content and research methods.

By fully searching the clinical database of our admitted patients, a total of three cases were included in the study that met the diagnostic criteria of TLC with pathological confirmation. Clinical data and photographs were obtained and desensitized for the protection of patients’ privacy. All the three patients had previously undergone tissue biopsy for the differential diagnosis of the lesions. The biopsy tissues were fixed in formaldehyde, embedded in paraffin, and preserved in the Department of Pathology of Fourth Medical Center of PLA General Hospital. With the patients’ consent, the specimens were obtained and sliced for further immunohistochemical staining.

### Immunohistochemical staining

For the obtained specimens, sections were incubated with DNase-free proteinase K (20 μg/ml; P1120, Solarbio, China) at 37°C for 30 min for antigen retrieval. Then, sections were incubated with 0.1% Triton X-100 (HFH10, Invitrogen, USA) in phosphate buffered saline (PBS) for 30 min to penetrate cell membrane. After blocking with 5% goat serum (16210064, Gibco, USA) in PBS for 30 min, the sections were incubated with corresponding antibodies overnight at 4°C. After incubation, sections were rinsed three times in PBS for 5 min each and then incubated with corresponding goat anti-rabbit or goat anti-mouse secondary antibodies conjugated with horseradish peroxidase for 1 h at room temperature. Sections were washed with PBS and then exposed to 3,3′-diaminobenzidine (DAB) staining (DA1016, Solarbio, China) following the manufacturer’s instructions. Finally, the slices were observed and photographed using a panoramic scanner from 3DHISTECH CaseViewer.

### Literature review and cases retrieval

A comprehensive literature review was conducted by searching the publications in PubMed (http://www.ncbi.nlm.nih.gov) and Embase (https://www.embase.com/) published between 1977 and 2022. The search terms used were “trichilemmal carcinoma”, “tricholemmal carcinoma”, and “tricholemmocarcinoma”. References not indexed in PubMed or Embase were also traced for a complete record. Specifically, the Cochrane Library (https://www.cochranelibrary.com/) and Prospero (https://www.crd.york.ac.uk/prospero/) were explored to obtain the TLC-relevant meta-analyses. The databases of ClinicalTrials.gov (https://clinicaltrials.gov/) and National Cancer Institute (https://www.cancer.gov/) were searched for any clinical trials on TLC. Translation of articles in languages other than English was executed via Google Translate (http://translate.google.com). The cutoff date was 7 July 2022. Eighty-six publications worldwide were identified from PubMed and Embase containing a total of 228 cases. No TLC-relevant meta-analyses or clinical trials were identified.

### Statistical analysis

Excel and SPSS were utilized for processing information. Missing values were ignored. Descriptive statistics were utilized. For categorical variables, frequencies and the percentage were computed, and Chi-square test or two-sided Fisher’s exact tests were performed. Continuous variables were first tested for normality by the Kolmogorov–Smirnov test and then for homogeneity by the variance test. Normally distributed data were presented as mean ± standard deviation and tested using t test. Non-normally distributed data were presented as the median and interquartile range and compared using the Mann–Whitney U-test. Covariates with a P-value < 0.05 in the univariate analysis were chosen for the multivariate analysis, where logistic regression was used to determine each variable with the recurrence of TLC. The 0.05 level of confidence was accepted as a significant difference.

## Results

### Case series

#### Case 1

**Figure 1:**
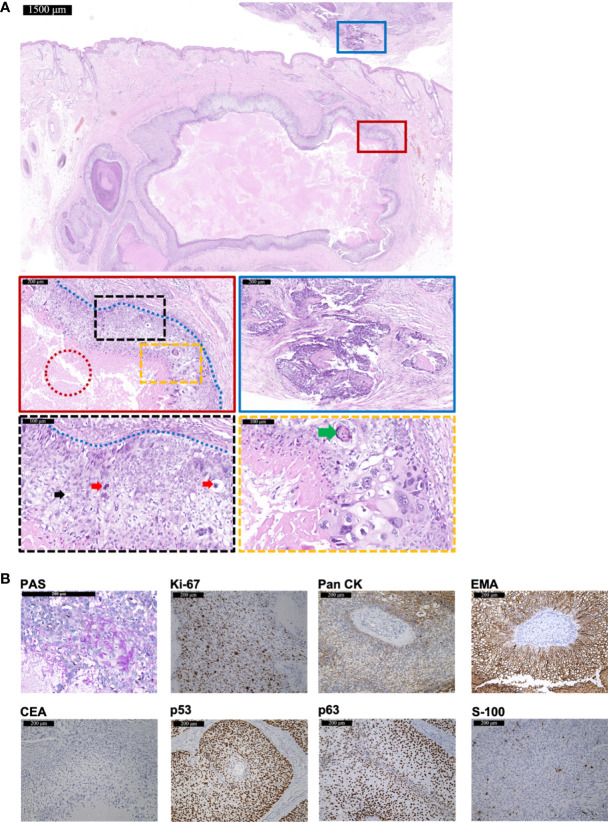
The histopathological and immunohistochemical features of Case 1 diagnosed as TLC. **(A)** The histopathological features of the surgical resection specimen of Case 1. The scale bar of the pathological overview picture is 1,500 μm, and the scale bars of the enlarged field of view are 200 and 100 μm, respectively. The pathological features in the solid and dashed boxes have been further zoomed in and displayed. The red dashed circle indicates the focal necrotic area visible within the tumor. The black dashed box shows mitosis (red arrows) and a large number of polygonal, transparent tumor cells (black arrows). The yellow dashed box shows a typical histopathology of trichilemmal keratinization (green arrows) and cellular atypia. The blue dashed line indicates the peripheral palisading of clear cells. The blue solid box shows the tumor invasion. **(B)** The staining features of Case 1. Case 1 showed positivity for PAS staining. As for immunohistochemical staining, Case 1 showed positivity for Ki-67 (60%+), Pan CK, EMA, p53, and p63 but negativity for CEA and S-100. The scale bar is 200 μm.

A 48-year-old man presented with a lesion on right temporal without itching, pain, or ulcers for 8 months. The 1.5-cm exophytic lesion was red and soft with multiple dilated capillaries on the surface, without pruritus or fluctuation. Any past medical history or hereditary disease were denied. Surgical excision was performed for further diagnosis. Hematoxylin-eosin (HE) staining revealed the infiltrative growth of the lesion (**Figure 1A**). The cytoplasm of tumor cells was clear (indicated by black arrows) and PAS-positive (**Figure 1B**), accompanied by scattered atypical nuclei and high mitotic index (indicated by red arrows). The blue dashed line indicated the peripheral palisading of clear cells. The typical histopathology of trichilemmal keratinization was indicated by the green arrows. In addition, focal necrotic area was visible within the tumor (indicated by red dashed circle). Immunohistochemistry showed positivity for Pan cytokeratin (Pan CK), EMA, Ki-67 (60%+), p53, and p63 but negativity for carcinoembryonic antigen (CEA) and S-100 (**Figure 1B**). For tumor cells seen at the bottom margins, further extended resection was performed and achieved tumor-free margins. A 54-month follow-up showed no recurrence or metastasis.

#### Case 2

**Figure 2:**
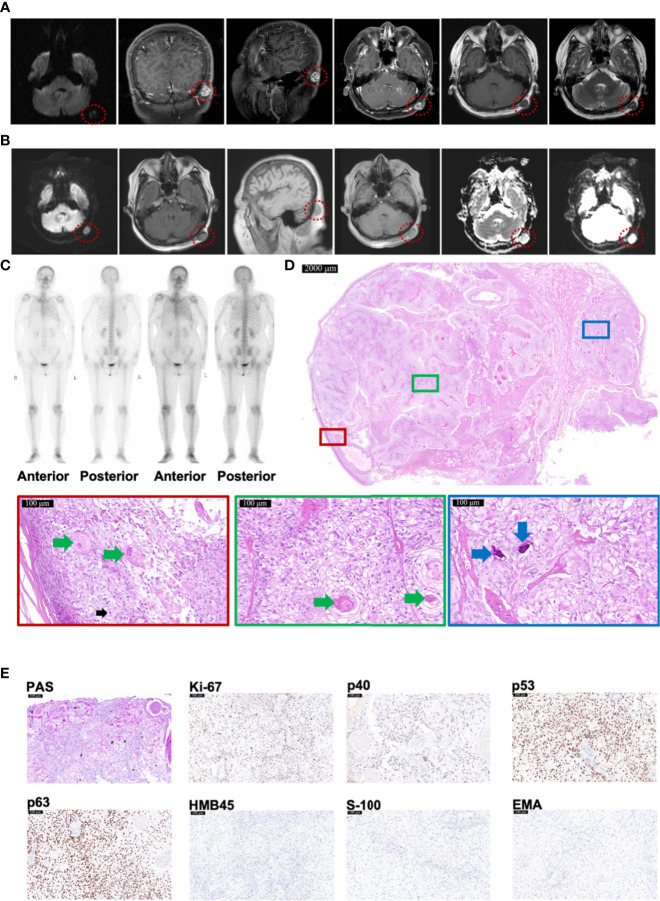
Radiology and histopathological features of Case 2 diagnosed as TLC. **(A, B)** MRI images of Case 2, taken respectively at her 57 **(A)** and 58 **(B)** years old, showing TLC nodules in the occipital region inside the red dotted circle. **(C)** The Emission Computed Tomography image of Case 2 showed that the bones of the whole body were not invaded by any tumor. **(D)** The histopathological features of Case 2. The scale bar of the pathological overview picture is 2,000 μm, and the scale bar of the enlarged field of view is 100 μm. The pathological features in the solid boxes have been further zoomed in and displayed. The lesion had many polygonal transparent tumor cells (indicated by black arrows), trichilemmal keratinization (indicated by green arrows), and calcification (indicated by blue arrows). **(E)** The staining features of Case 2. Case 2 showed positivity for PAS staining. As for immunohistochemical staining, Case 2 showed positivity for Ki-67 (35%), p53, and p63 but negativity for p40, HMB45, S-100, and EMA. The scale bar is 100 μm.

A woman suffered invasive ductal carcinoma of left breast with vessels infiltrated at age 55. Two years after the resection, head magnetic resonance imaging (MRI) detected an abnormal signal of 1.7 × 1.2 cm in left occipital subcutaneous without invasion to the skull or brain (**Figure 2A**). Another year later, MRI reported an abnormal signal of 1.8 × 1.3 cm in left occipital subcutaneous, whose imaging appearance was similar to that of 1 year ago (**Figure 2B**). No bone metastases were found by emission computed tomography (CT) (**Figure 2C**). After resection, pathological examination revealed that the tumor was attached to the epidermis with 2.2 × 2.0 × 1.5 cm in size (**Figure 2D**). The lesion had a classic presentation of TLC with many polygonal transparent tumor cells (indicated by black arrows), trichilemmal keratinization (indicated by green arrows), and calcification (indicated by blue arrows) (**Figure 2D**). PAS staining was positive (**Figure 2E**). Immunohistochemistry staining showed positivity for Ki-67 (35%), p53, and p63 but negativity for p40, HMB45, S-100, and EMA (**Figure 2E**). All resection margins were negative. A 24-month follow-up showed no recurrence.

#### Case 3

**Figure 3:**
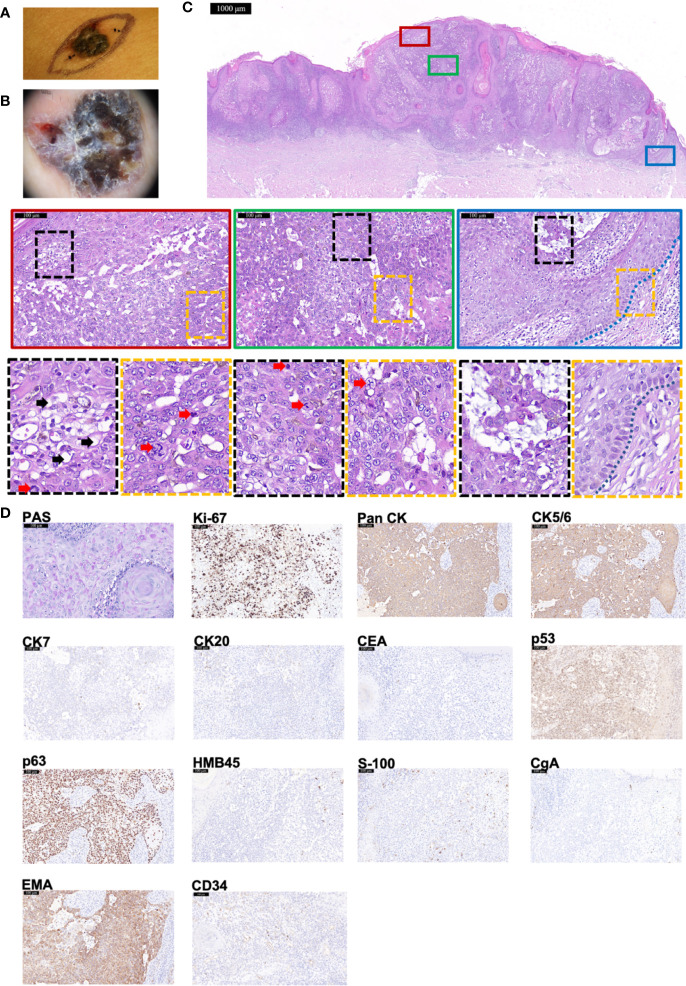
The histopathological and immunohistochemical features of Case 3 diagnosed as TLC. **(A, B)** Macroscopic and dermoscopic view of the TLC lesion of Case 3. **(C)** The histopathological features of Case 3. The scale bar of the pathological overview picture is 1,000 μm, and the scale bar of the enlarged field of view is 100 μm. The pathological features in the solid and dashed boxes have been further zoomed in and displayed. The lesion had many polygonal transparent tumor cells (indicated by black arrows) and pathological mitosis (indicated by red arrows). The blue dashed line indicates the peripheral palisading of cells. **(D)** The staining features of Case 3. Case 3 showed positivity for PAS staining. As for immunohistochemical staining, Case 3 showed positivity for Ki-67 (80%+), Pan CK, CK5/6, p53, p63, and EMA but negativity for CK7, CK20, CEA, HMB45, S-100, and CgA. CD34 was diffusely expressed. The scale bar is 100 μm.

An 84-year-old woman presented with a dark-brown papule on right breast, with rough surface, uneven pigmentation, and clear boundaries (**Figure 3A**). Dermoscopy showed the nodule was an asymmetrical, multi-lobed lesion with crystalline pupa-like structures distributed (**Figure 3B**). There was no palpable lymphadenopathy. After the initial diagnosis of suspicious BCC, wide local excision was executed. HE staining manifested several features supporting a diagnosis of TLC—predominantly clear cytoplasm within the tumor cells (indicated by black arrows), many cells in mitosis (indicated by red arrows), and focal peripheral palisading (indicated by blue dashed line) (**Figure 3C**). PAS staining was positive (**Figure 3D**). Immunohistochemistry staining (**Figure 3D**) showed positivity for Ki-67 (80%+), Pan CK, EMA, CK5/6, p53, and p63 but negativity for CEA, CgA, CK7, CK20, HMB45, and S-100. In addition, CD34 was diffusely expressed. All of these supported the diagnosis of TLC. At 6-month follow-up, there were no signs of recurrence.

### Global trends and regional distribution

**Figure 4:**
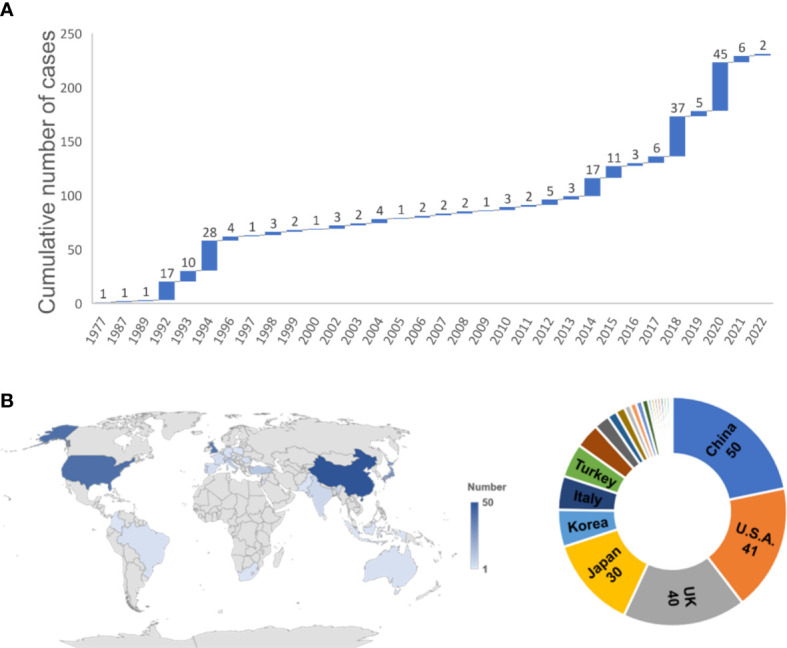
Temporal and spatial distribution of 231 TLC cases. **(A)** The annual and cumulative number of TLC cases shown in a waterfall chart from 1977 to 2022. **(B)** Regional distribution of the 231 TLC cases worldwide.

All the reported cases were collected to analyze the characteristics of TLC. In total, 231 cases (including three cases in this article) were included. Because of the infrequent reporting or tracking of TLC occurrences, the actual incidence could not be accurately determined. The cumulative number of cases has grown slowly since the mid-1990s but been increasing rapidly since 2014 with a gradually upward growth curve (**Figure 4A**). The year 2020 (45, 19.48%), 2018 (37, 16.02%), and 1994 (28, 12.12%) were the top three years with the highest volume of published TLC cases (**Figure 4A**). All continents except Antarctica have cases, with most of the cases located in Asian (112, 48.48%) (**Figure 4B**). China ranked first (50, 21.65%), followed by the USA (41, 17.75%), UK (40, 17.32%), Japan (30, 12.99%), and Korea (12, 5.19%).

### Demographic characteristics

The clinical demographic information of the 231 cases is summarized in **Table 1**. A total of 121 cases were men, slightly more than 110 of women (**Table 1**). The age ranged from 9 to 95 years, and there was no statistical difference between genders (t = 1.623, P = 0.1064). However, when grouped by every 20 years, the age distribution between genders was inconsistent (c^2^ = 11.255, P = 0.016). The age group with the highest proportion of men was 61–80 with 51 cases (53.68%), higher than that of the women (24 cases, 32.43%, P < 0.05). Meanwhile, the proportion of female patients over 80 years old was the highest (31 cases, 41.89%), which is statistically different from that of the male patients (19 cases, 20.00%, P < 0.05).

**Table 1:** The demographic information of 231 TLC cases.

	Total (n = 231)	Male (n = 121)	Female (n = 110)	t/c^2^/Z-value	*P*-value
Age, years, mean ± SD	65.70 ± 16.45	66.85 ± 16.05	71.18 ± 18.53	1.623	0.1064
0–20, mean (%)	2 (1.18)	1 (1.05)	1 (1.35)	11.255	0.016
21–40, mean (%)	12 (7.10)	7 (7.37)	5 (6.76)		
41–60, mean (%)	30 (17.75)	17 (17.89)	13 (17.57)		
61–80, mean (%)	75 (44.38)	51 (53.68)	24 (32.43)*		
81–100, mean (%)	50 (29.59)	19 (20.00)	31 (41.89)*		
Location, Freq (%)					
Head and Neck	194 (83.98)	99 (81.82)	95 (86.36)	8.288	0.508
Leg	15 (6.49)	7 (5.79)	8 (7.27)		
Chest	6 (2.60)	4 (3.31)	2 (1.82)		
Hand	5 (2.16)	4 (3.31)	1 (0.91)		
Arm	2 (0.87)	0 (0.00)	2 (1.82)		
Abdomen	2 (0.87)	1 (0.83)	1 (0.91)		
Shoulder	2 (0.87)	2 (1.65)	0 (0.00)		
Armpit	2 (0.87)	2 (1.65)	0 (0.00)		
Buttocks and perineum	2 (0.87)	1 (0.83)	1 (0.91)		
Back	1 (0.43)	1 (0.83)	0 (0.00)		
Diameter, cm, M (*P*25, *P*75)	2.00 (1.00, 4.08)	2.00 (1.00, 4.88)	2.00 (1.28, 3.50)	0.442	0.658
Thickness, cm, M (*P*25, *P*75)	2.25 (0.78,4.13)	2.50 (2.00, 5.25)	0.80 (0.50, 3.50)	2.106	0.035
Duration, months, M (*P*25, *P*75)	13.0 (6.0, 36.0)	12.0 (5.0, 25.5)	24.0 (6.0, 42.0)	1.525	0.127
Regional lymph node metastasis, Freq (%)	9 (3.90)	5 (4.13)	4 (3.64)	0.000	1.000
Carcinoma history, Freq (%)	10 (4.33)	4 (3.31)	6 (5.45)	0.228	0.633
Recurrence, Freq (%)	19 (8.23)	12 (9.92)	7 (6.36)	0.964	0.326
Follow-up, months, M (*P*25, *P*75)	24.00 (12.00, 48.00)	24.00 12.0, 40.75)	24.00 (15.00, 60.00)	1.207	0.228

Asterisks indicated a statistically significant difference in the ratio of male and female patients in that age group. M, Median.

Most common location was the head and neck (194 cases, 83.98%). The remaining cases were distributed on leg, chest, hand, arm, abdomen, shoulder, armpit, buttocks and perineum, and back. There was no statistical difference in the distribution between genders (c^2^ = 8.288, P = 0.508).

### Clinical features

The phenotype of TLC was not always typical. In addition, TLC may be misdiagnosed or overlooked due to the co-occurrence of other diseases. Three cases presented with actinic keratosis simultaneously, two cases on ear, and one case on temple (4–6). The lesion on the abdomen of a 76-year-old woman was diagnosed as TLC arising in seborrheic keratosis (7). A case of TLC in facial sebaceous glands was accompanied by Syringocystadenoma papilliferum (8). There were also cases of TLC with primary cutaneous anaplastic large-cell lymphoma, a ductal eccrine component, nevus sebaceous, or a proliferating trichilemmal cyst (9–12). Some cases were combined with other carcinoma at the lesions, four cases with BCC (13, 14), two cases with Bowen’s disease (15, 16), and one case with Merkel cell carcinoma (17).

Manifestations of TLC ranged in diameter from 0.3 to 37 cm, and the thickness ranged from 0.30 to 10.00 cm. Diameter was not statistically different between genders (Z = 0.442, P = 0.658). On the contrary, male cases appear to have superiority in thickness (median, 2.50 cm) than that of female cases (median, 0.80 cm, Z = 2.106, P = 0.035). The duration had a median time of 13.0 months; 12.0 months in men and 24.0 months in women but with no statistical difference (Z = 1.525, P = 0.127).

**Figure 5:**
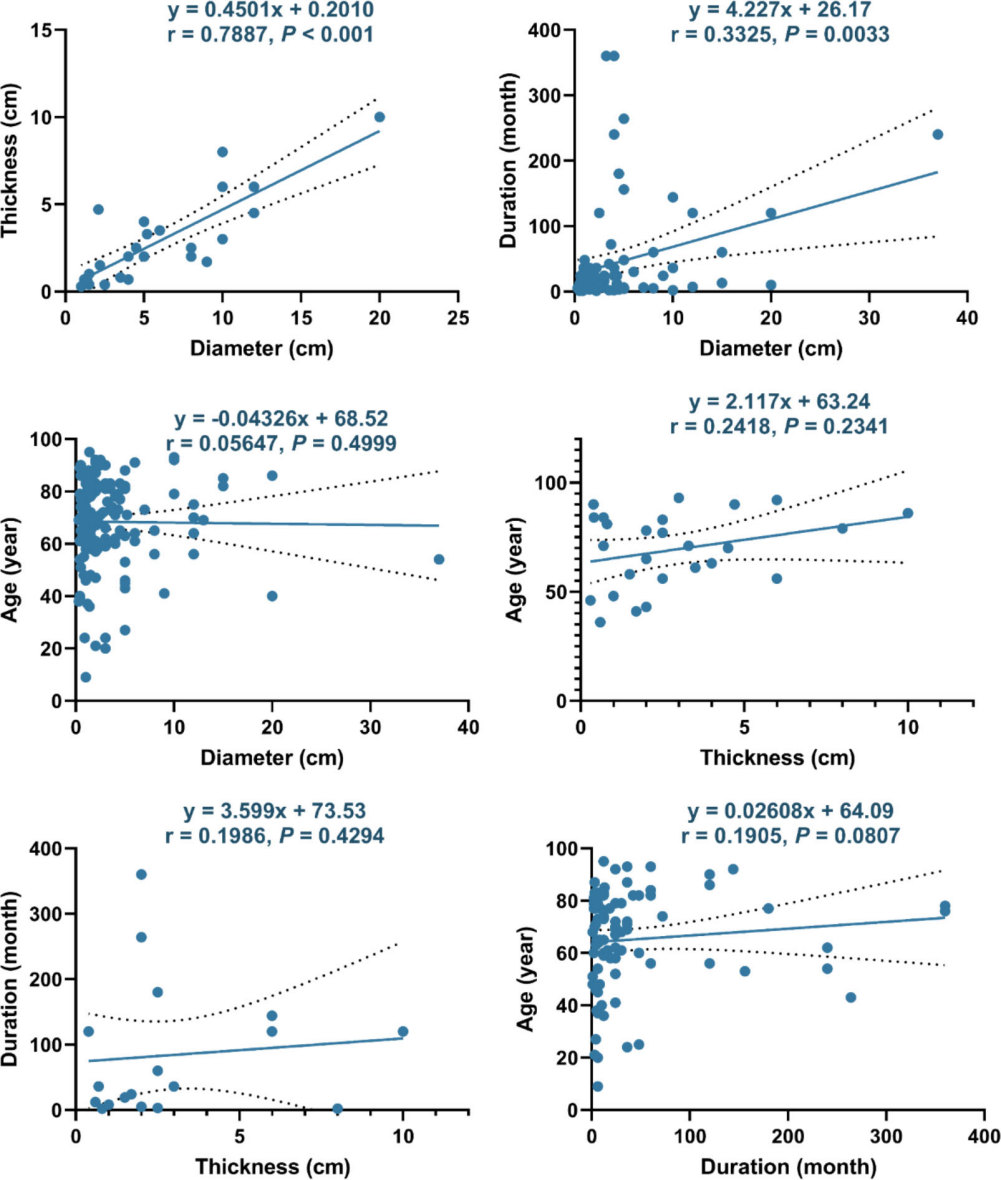
Linear correlations between different factors of TLC. The dotted line represents the 95% confidence interval.

Furthermore, we explored factors that may be related to the size or duration of TLC (**Figure 5**). The diameter of TLC had an obvious linear relationship with its thickness (r = 0.7887, P < 0.001) and a relatively weak linear relationship with the duration (r = 0.3325, P = 0.0033) but had no linear relationship with the age (r = 0.0565, P = 0.4999). In addition, no linear relationship was found between thickness with age (r = 0.2418, P = 0.2341), thickness with duration (r = 0.1986, P = 0.4294), or duration with age (r = 0.1905, P = 0.0807).

Another thing to note is that nine cases were accompanied by regional lymph node metastasis, with five men and four women.

### Pathogenesis

The pathogenesis of TLC has not been clearly elucidated. A total of 87.01% of TLCs (201 of the 231 cases) occurred in the sun-exposed sites (head, neck, arm, and hand), 6.7 times of the non-exposed sites, suggesting the significant role of UV in the pathogenesis of TLC. Ten cases (4.33%) had previous carcinoma: four cases with breast cancer (including Case 2) (18–20), five cases with BCC (two of whom also had SCC) (13,) (21–24), and one case with both SCC and Bowen’s disease (25). There were four and six cases in men and women, respectively (c^2^ = 0.228, P = 0.633). Moreover, locally present skin lesions, trauma, or scarring may also be associated with the progression of TLC. Two cases were secondary to a previous site of actinic keratosis in neck and forehead, respectively (13, 25). TLC was also secondary to a previous proliferating trichilemmal (26), psoriasis (21), or cutaneous horn of the location (27). Local inappropriate physical or chemical stimulation may trigger the occurrence of TLC. A 36-year-old woman developed TLC after receiving laser treatment in the postauricular area (28). Prolonged topical use of hair removal cream on armpits also triggered TLC, with metastases to regional lymph nodes (29). In two cases, TLC was secondary to scars caused by burns 5 years or even 42 years ago (30). Ionizing radiation could induce carcinogenesis by inducing DNA damage and gene mutation. A 67-year-old man, who had been exposed to multiple x-rays (50–60 times in total) and CT of the chest, developed a 1.5-cm TLC with regional recurrence and distant metastasis after resection (31). However, it should be noted that the role of these factors in the occurrence and development of TLC may only be hypothesized now and remains to be further determined, given that the number of related cases was all small, which could very well be a random event.

Studies have shown that advanced age, Caucasian, male sex, and a chest organ transplant are the risk factors for skin carcinoma after solid organ transplantation (32). Here, a total of four cases from 39 to 63 years underwent transplantation [two kidney transplants (33, 34), one heart transplant (35), and one unknown (36)], all were men with three Caucasians and one Asian. Because immunosuppression may affect the phenotype of TLC, the true incidence of TLC secondary to solid organ transplantation may be higher (37).

Additional risk factors of TLC also include genetic disorders. As an autosomal recessive genetic skin disorder, patients with xeroderma pigmentosum (XP) have impaired DNA repair ability (38, 39). A 25-year-old man with XP presented with TLC in cheek and eyelid (40). Another 9-year-old girl with XP also suffered from TLC in the nose (41). Both cases were much younger than the average age, suggesting XP as a strong risk factor for TLC. The impaired DNA repair ability of XP was considered to be related to the changes of p53 to some extent (42). As a key anti-cancer protein with growth inhibitory function, p53 can regulate the cell cycle and promote cell apoptosis or senescence, thus inhibiting tumorigenesis (43). Studies have found that the total loss of chromosome arm 17p (where the TP53 gene resides) was found in a TLC case, leading to the blocked expression of p53, which may be associated with the marked increase of the proliferation fraction and thus contribute to the development of TLC (12).

### Histopathological features

With the same tissue origin as trichilemmoma, TLC has similar histologic features but varies by atypical nuclei and high mitotic index (44). Centered on a pilosebaceous unit, TLC generally presents a typical histopathology of trichilemmal keratinization, a peripheral palisading pattern and focal necrosis (41, 45). The growth of TLC always presents lobular and invasive, characterized by classical large, polygonal, transparent tumor cells with eccentric nuclei but without hair follicle differentiation (41, 46). In some cases, TLC was found attached to the epidermis and infiltrating the hair follicle within the dermis, with the infiltrative lobules of clear cells where trichilemmal keratinization can be found (47).

In general, the pathological diagnostic criteria of TLC are not uniform and varied among different authors and doctors, but the following criteria for diagnosing are accepted and recognized by the majority: (1) PAS-positive glycogen within neoplastic cells, (2) folliculocentricity, (3) peripheral palisading of clear cells, (4) a prominent Periodic Acid Schiff Diastase (D-PAS)–positive basement membrane, (5) trichilemmal keratinization, (6) lobular architecture, and (7) the presence of pre-existing trichilemmoma (30, 48). TLC may be confused with other skin cancer, such as SCC with clear cell differentiation, BCC with keratin cysts and peripheral palisading cells within the basaloid islands, or even the keratoacanthoma, which was benign and could resolve spontaneously (27, 49–51). Given the similarity of cell origin and pathological phenotype, the accurate diagnosis of TLC from other tumors originated from the skin and the adnexa may sometimes be difficult and relies further on stains other than HE staining.

**Figure 6:**
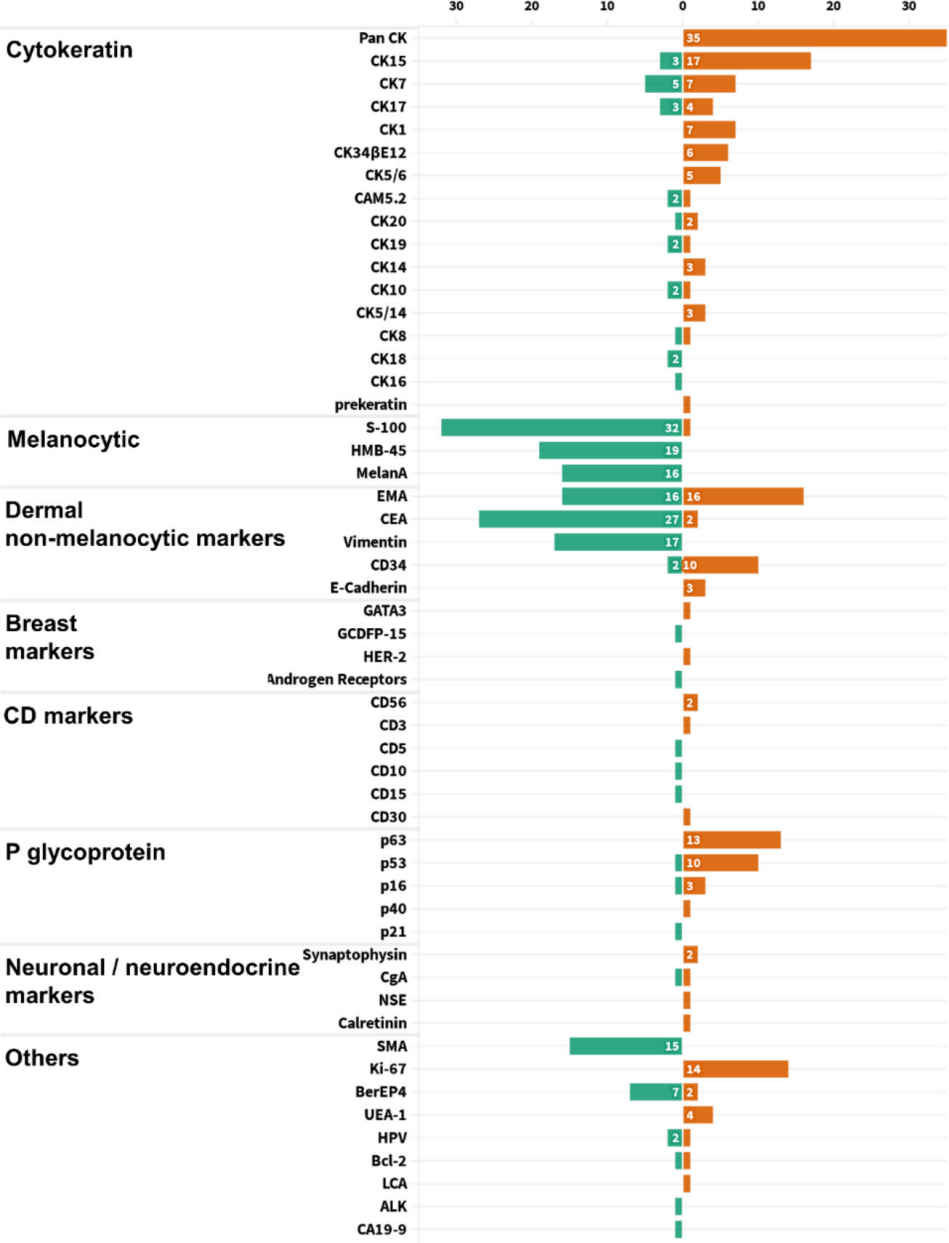
Statistical chart of pathological indicators used in all 231 TLC cases, divided into eight categories for display.

**Figure 7:**
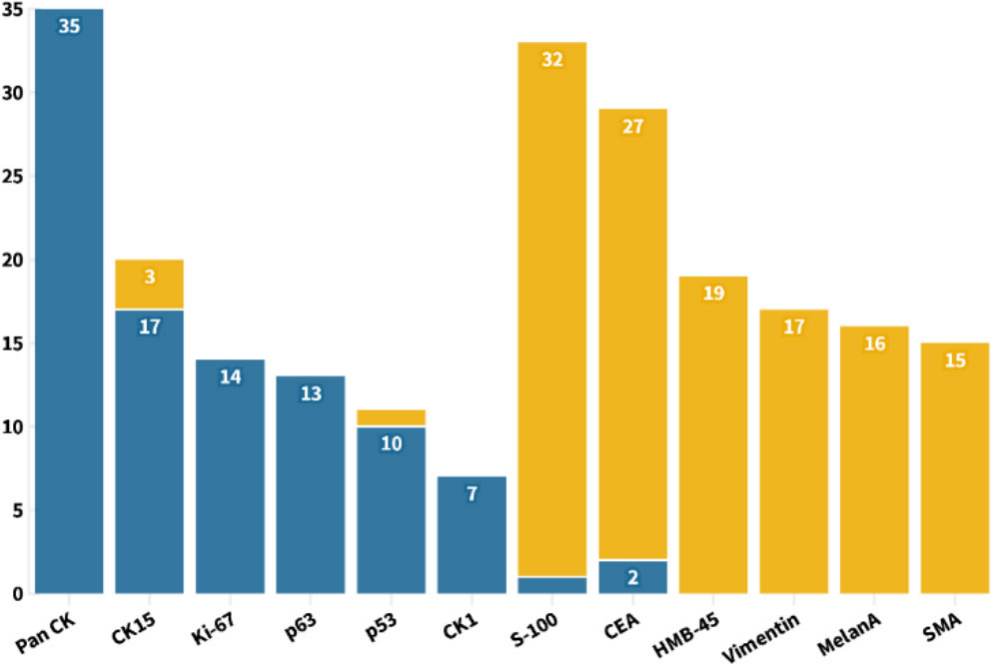
With the frequency no less than 7 as the screening criteria, the six pathological indicators with the highest positive and negative rates were shown for the diagnosis of TLC.

In general, stains were not performed unless the differential diagnosis from other mimickers was necessary. From the statistics of all published cases, PAS staining was used in 73 cases, all of which were positive. Mucicarmine staining was applied in 23 cases, and all samples were negative. All the other 53 pathological indicators were counted and sorted into eight categories, with an average frequency of 7.06 (**Figure 6**). Using the frequency ≥ 7 as filter criterion, there were 17 remained indicators. Furthermore, the top six indicators of positive and negative rates, respectively, are listed in **Figure 7**. It is worth noting that Pan CK ranked first with a frequency of 35 times, but its differential diagnostic value for skin tumors with clear cell changes was limited, because Pan CK was always positive in the mimickers, except for tumors arising from different lines of differentiation, such as melanoma, which, in turn, may not be difficult to diagnose due to its distinct appearances in HE staining (52).

As a glycoprotein localized on the surface of cell membrane, CD34 is involved in the adhesion of cell–cell and cell–extracellular matrix (53). In addition to being used as a marker of hematopoietic stem cells, CD34 is also a clue to the differentiation from the outer root sheath and follicular, which is of great significance for the diagnosis of TLC (54–56). Here, a total of 12 cases of TLC were stained for CD34, of which 10 cases (83.33%) were positive. The staining of CEA in TLC was usually negative, and, of the 29 cases, only two cases were positive (**Figure 7**). Moreover, EMA was widely adopted with a frequency up to 32 times, with half positive and half negative, suggesting that EMA may not be a suitable marker for identifying TLC (**Figure 6**).

### Treatment and prognosis

**Figure 8:**
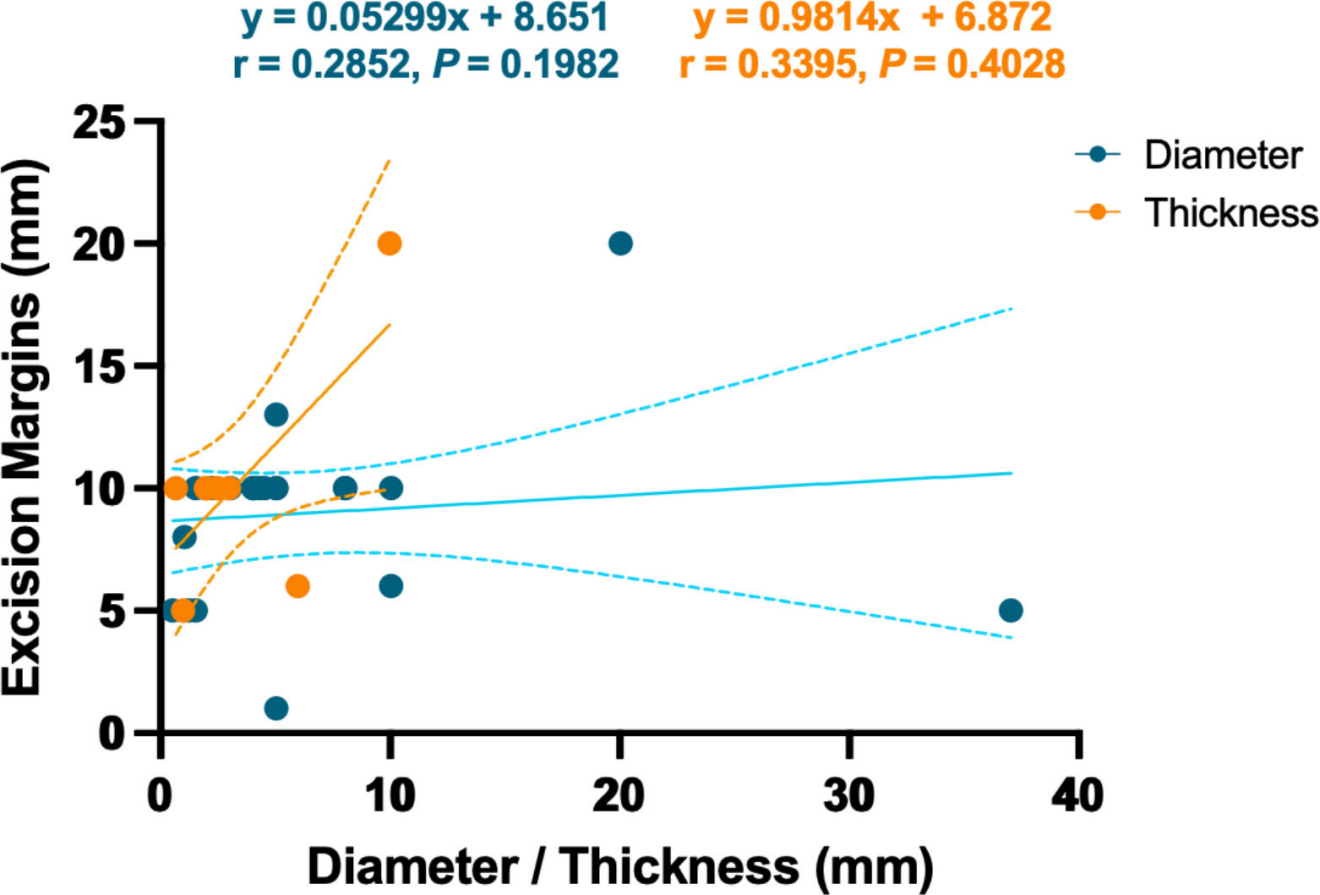
Linear relationship between the excision margin and the diameter or thickness of the TLC. The dotted line represents the 95% confidence interval.

A total of 212 cases were treated by surgical excision, among which 16 cases (7.55%) experienced a recurrence. Mohs micrographic surgery (MMS) has been widely used in the resection of carcinoma especially in the head and face (57, 58). Sixteen cases of TLC were resected by MMS, of which two cases had tumor recurrence, namely, 77-year-old man and 65-year-old man both in cheek (23, 59). The recurrence rates of surgical excision and MMS were not statistically different (c^2^ = 0.052, P = 0.820). Another recurrence occurred in a 64-year-old man who refused treatment (60). Excision margins ranged from 1 to 20 mm, with no linear relationship with TLC diameter or thickness (r = 0.2852 and 0.3395, P = 0.1982 and 0.4028, **Figure 8**). As for MMS, there was only one documented resection margin, which was 8 mm.

Other interventions have also been reported. Imiquimod cream (5%) successfully healed a 90-year-old woman with a lesion of 2.5 × 2.0 × 0.39 cm on the cheek (61). Chemotherapy was used to treat a patient with TLC with primary cutaneous anaplastic large-cell lymphoma (9). Three cases underwent additional radiotherapy after excision (62, 63). Two cases received both chemotherapy and radiotherapy after excision. A 27-year-old man successfully relieved his 5-cm lesion on the neck (26). However, the lesion in 64-year-old man’s leg still recurred after treatment (58).

Nineteen cases were recorded with recurrence of TLC. We further analyzed the risk factors for TLC recurrence with cases divided into recurrence or non-recurrence groups (**Table 2**). There was no statistically difference in sex, age, location, thickness, regional lymph node metastasis, or regional non-carcinoma lesions (P > 0.05). Diameters and duration were much higher in the recurrence group (Z = 2.711 and 2.073, P = 0.007 and 0.038), with carcinoma history more frequently occurred (c^2^ = 4.992, P = 0.025). Further logistic regression analysis revealed that carcinoma history was the independent risk factors for the recurrence of TLC, with odds ratio (OR) of 5.448 and 95% CI of 1.428–20.778 (P = 0.013, **Table 3**).

**Table 2:** Statistical information on risk factors associated for TLC recurrence.

Characteristics	Recurrence (n = 19)	Non-recurrence (n = 150)	t/c^2^/Z-value	*P*-value
Male, Freq (%)	12 (63.16)	83 (55.33)	0.419	0.517
Age, years, mean ± SD	64.84 ± 17.95	69.24 ± 17.17	1.103	0.27
0–20, mean (%)	20 (5.26)	9(0.67)	0.35	0.726
21–40, mean (%)	35 (5.26)	31.91 (7.33)		
41–60, mean (%)	46 (10.53)	53.82 (18.67)		
61–80, mean (%)	62.75 (57.89)	70.77 (42.67)		
81–100, mean (%)	85.75 (21.05)	86.74 (30.37)		
Location, Freq (%)			7.525	0.529
Head and Neck	13 (68.42)	119 (79.33)		
Leg	3 (15.79)	12 (8.00)		
Chest	2 (10.53)	4 (2.67)		
Hand	1 (5.26)	4 (2.67)		
Arm	0 (0.00)	2 (1.33)		
Abdomen	0 (0.00)	2 (1.33)		
Shoulder	0 (0.00)	2 (1.33)		
Armpit	0 (0.00)	2 (1.33)		
Buttocks and perineum	0 (0.00)	2 (1.33)		
Back	0 (0.00)	1 (0.67)		
Diameter, cm, M (*P*25, *P*75)	4.00 (1.88, 5.80)	2.00 (1.00, 4.00)	2.711	0.007
Thickness, cm, M (*P*25, *P*75)	3.40 (1.05, 3.88)	2.00 (0.78, 4.55)	0.285	0.776
Duration, months, M (*P*25, *P*75)	5 (3.5, 11.25)	18 (6, 42)	2.073	0.038
Regional lymph node metastasis, Freq (%)	1 (5.26)	8 (5.33)	0.000	1.000
Carcinoma history, Freq (%)	4 (21.05)	7 (4.67)	4.992	0.025
Regional non-carcinoma lesions, Freq (%)	4 (21.05)	22 (14.67)	0.152	0.697

M, Median.

**Table 3:** Analysis of independent risk factors for TLC recurrence.

Variable	Odds ratio	95% CI	*P*-value
Duration	0.925	0.831–1.029	0.151
Diameter	1.048	0.960–1.144	0.295
Carcinoma history	5.448	1.428–20.778	0.013

## Discussion

Starting from three clinical cases, we conducted a comprehensive statistical analysis of TLC, which will help us in the diagnosis and treatment of TLC. From the perspective of susceptible population, the majority of patients with TLC are men aged 60–80 and women over 80. The specific reason for this age distribution difference is unclear, probably owing to the inconsistent lifestyles of men and women, in that women generally pay more attention to sun protection. It is worth noting that patients as young as 9 years old also have the possibility of TLC, especially when they have genetic disorders, local trauma, or other skin lesions. Moreover, radiotherapy for previous tumors may lead to the occurrence of TLC. Given the progressive increase in the incidence of TLC recently, dermatologists should be alert to the possibility of TLC when encountering adnexal skin tumors, especially those located on head and neck.

TLC has diverse and, sometimes, confusing clinical manifestations and occasionally may not be correctly diagnosed (64). Partly because of the advanced age, some patients’ TLC was accompanied by a variety of other skin diseases from actinic keratosis to BCC, suggesting that doctors should fully consider all possibilities when making diagnosis. As a rare adnexal carcinoma, TLC was not generally considered as an aggressive tumor. However, the longer duration of TLC generally represents a larger diameter, which, in turn, represents a deeper infiltration and, in some cases, manifested as locally aggressive or metastasis to regional lymph nodes (51). In cases with longer course of disease, dermatologist should explore the draining lymph nodes, and the resection margins should be surely free of tumor cells and carefully observed for infiltration.

Nowadays, the differential diagnosis of TLC from other diseases has mainly relied on HE staining. PAS staining was also commonly employed. However, we cannot rely on PAS staining for making the diagnosis of TLC. It can also be positive in clear-cell SCC or clear-cell BCC because it highlights glycogen (65, 66). Special stains can be additionally suggestive clues when differential diagnosis is difficult.

Compared with TLC, clear-cell SCC lacks trichilemmal keratinization, lobular proliferation, peripheral palisading, and pushing border. Moreover, clear-cell BCC lacks keratinization of outer root sheath. Balloon cell melanoma was positive for S-100 and HMB-45 staining, and the melanin pigment was positive to the Masson-Fontana reaction. Hidradenocarcinoma has ductal differential with CEA and EMA staining. Sebaceous carcinoma may present with multiple intracytoplasmic lipid-rich vacuoles indenting the nucleus, with positive adipophilin. Malignant proliferating trichilemmal tumor is synonymous with proliferating trichilemmal cystic carcinoma and has cystic structure and intercellular bridge. Metastatic clear-cell adenocarcinomas of the viscera were generally renal-derived or ovarian-derived. Renal-derived clear-cell carcinoma was commonly positive for CD10, PAX8, and Vimentin (67). In addition, ovarian-derived clear-cell carcinoma was positive for HNF1-β and Napsin A (68, 69).

After statistical analysis of all cases, we propose that the positivity of Pan CK, CK15, Ki-67, p63, p53, and CK1 and the negativity of S-100, CEA, HMB-45, Vimentin, MelanA, and SMA may be constructive for the diagnosis of TLC. Furthermore, the positive expression of TLC on CK1, CK10, CK14, and CK17 indicates that TLC differentiated toward follicular infundibulum, which may have implications for expanding the use of follicular infundibulum–like cells as a diagnostic clue for TLC. Indicating the differentiation from the outer root sheath, CD34 is a valuable biomarker for the diagnosis of TLC with relatively a high positive rate. As for the expression of EMA in TLC, there is still no definite conclusion. EMA was considered to be positive in TLC in some dermatology textbooks, like *Textbook of Dermatopathology* by Prof. Raymond L. Barnhill. However, *McKee’s Pathology of the Skin* by Prof. Eduardo Calonje et al., *China Clinical Dermatology* by Prof. Bian Zhao, and *Practical Dermatopathology* by Prof. Tianwen Gao et al. hold the view that EMA is usually negative in TLC. On the basis of the statistical results of all published TLC cases, in total, the positive rate of EMA was 50% and thus may not be meaningful for TLC’s differential diagnosis (70). It should be noted that the pathological criteria for diagnosing TLC varied among different authors. Meanwhile, given the similar cellular origin and pathological phenotype between TLC and other skin tumors like SCC, some dermatologists and pathologists also hold the view that TLC may be clear-cell SCC, a subtype of SCC but not a special disease (66, 71). In addition, some doctors concluded that the tumor is very rare if it exists at all and may be over diagnosed by its adherents. Further conclusions urgently need more and larger pathological studies to summarize the unique pathological characteristics of TLC different from other skin tumors.

As most cases reported, TLC was managed with complete surgical resection (72). MMS has been proven to be an effective treatment for TLC, but it is still necessary to ensure that the surgical margins are free of tumor cells for it is not foolproof. For nodules larger than 5 cm in diameter, radiotherapy or chemotherapy can also be considered. For patients with extra carcinoma history, special care should be taken during excision and follow-up to avoid recurrence. We recommend patients to have follow-up between 6 and 12 months. During the period, patients should be advised to pay attention to sun protection and return for further consultation with new lesions.

## Data availability statement

The original contributions presented in the study are included in the article/supplementary material. Further inquiries can be directed to the corresponding author.

## Ethics statement

The studies involving human participants were reviewed and approved by Ethics Committee of Fourth Medical Center of PLA general hospital. The patients/participants provided their written informed consent to participate in this study.

## Author contributions

YY conceptualized the study and designed the experiments. JS and LZ wrote the manuscript. JS, MX, SL, and RC collected and analyzed the data. YL carried out the pathological staining required for article revision.
